# From Endoscopy to Empathy: Redefining Gastroenterology Training for the Gut-Brain Era

**DOI:** 10.14309/crj.0000000000001957

**Published:** 2026-01-07

**Authors:** Sweta Sahu, Hareesha Rishab Bharadwaj, Shahryar Khan, Hassam Ali, Dushyant Singh Dahiya

**Affiliations:** 1 Department of Medicine, J.J.M. Medical College, Davangere, Karnataka, India; 2 Faculty of Biology Medicine and Health, The University of Manchester, Manchester, UK; 3 Department of Internal Medicine, The University of Kansas Health System, Kansas City, KS; 4 Department of Gastroenterology, Hepatology and Nutrition, East Carolina University/Brody School of Medicine, Greenville, NC; 5 Division of Gastroenterology, Hepatology and Motility, The University of Kansas School of Medicine, Kansas City, KS

**Keywords:** gastroenterology, hepatology, medical education, brain-gut axis, functional disorders

Disorders of gut-brain interaction (DGBI, formerly functional gastroenterology [GI] disorders) are commonly encountered in clinical practice and pose a significant health burden to patients and the healthcare system. Recent population-based studies estimate that up to 40% of adults suffer from DGBI internationally, affecting their quality of life daily.^1,2^ However, these conditions remain under-recognized and inconsistently taught during GI fellowship training. A 2022 survey conducted in Great Britain reported that only irritable bowel syndrome (IBS) is consistently taught in the medical curricula, but functional GI disorders are largely under-taught in both undergraduate and subspecialty training.^2^ This gap contributes to a significant knowledge deficit among new gastroenterologists while managing DGBI.^1,3^

The aim of this editorial was to highlight the deficiencies in GI training for DGBI at national and international levels and to offer real-world solutions to bridge the gap and ensure comprehensive and empathetic training for future gastroenterologists.

Training Deficiencies and Trainee Attitudes: Training deficiencies shape trainee attitudes toward DGBI. In the United States (US), an alarming 40% of GI fellows report dismissive attitudes toward DGBI patients by supervisors and peers.^1^ Over 20% of these trainees often feel frustrated or burned out when they encounter DGBI cases, and by the end of fellowship nearly 40% actively avoid such patients in clinical practice.^1^ Those who see DGBI patients in clinic lack confidence in DGBI management, with only 32% feeling comfortable initiating neuromodulator therapy.^1^ Furthermore, less than one-third of these clinicians know when to involve GI behavioral specialists.^1^ Likewise, in the United Kingdom (UK), fewer than half of GI trainees report regular DGBI clinic exposure and most rarely feel comfortable making a DGBI diagnosis or prescribing the recommended treatment.^4^ These findings illustrate how insufficient training directly affects trainee confidence and preparedness.

The root cause of this training deficiency is primarily curriculum-driven. An Austrian national survey reported diagnosis of functional GI disorders to be the area with the largest room for improvement in clinical training.^5^ Similarly, many training programs do not include formal DGBI modules, only about 50% of trainees worldwide report that a structured curriculum exists at their institution.^3,5^ In 1 pilot study, although a dedicated didactic series on DGBI improved knowledge, it did not change attitudes, highlighting how entrenched gaps in training persist.^3^ The standard GI curriculum places a strong and recurrent emphasis on organic diseases of the GI tract and procedural skill during endoscopy with regular feedback to improve proficiency. However, limited attention is given to functional GI disorders.^2^ Hence, trainees become highly proficient in endoscopy and management of organic GI diseases while lacking confidence in managing the biopsychosocial complexity of DGBI. This gap is then perpetuated as trainees become supervising physicians, reinforcing cultural and educational shortcomings.

Global Perspectives on Gastroenterology Education: The training shortfalls are not unique just to the US or UK. These deficiencies are also observed globally, and international data offer similar findings. In Europe, the 2022 Rome Foundation census noted that IBS is the only DGBI consistently taught across medical training, with most other DGBI seldom covered in the curriculum.^2^ In the 2025 pan-European survey, department heads overestimated the quality of training, emphasizing the need for train-the-trainer initiatives to improve competency in diagnosing functional bowel disorders.^5^

In developing regions, program evaluations likewise call for substantial reform. A 2025 national survey of GI fellowship programs in Saudi Arabia (analogous to the US three-year model) found moderate overall satisfaction but significant variability, with only about 41% of trainees having dedicated didactic sessions and limited research opportunities.^6^ Respondents urged adoption of standardized curricula, inclusion of training across the full spectrum of GI disorders, stronger mentorship from seasoned gastroenterologists, and the incorporation of proven international models to produce well-rounded and competent specialists.^6^

The urgency of improving DGBI training has intensified in the post-COVID-19 era. A July 2025 international study reported that overall DGBI prevalence increased from 38% prepandemic to 42.6% in 2023, with prevalence of IBS and functional dyspepsia rising by 28% and 44%, respectively.^7^ Furthermore, current research suggests that patients with long COVID are significantly more likely to develop new DGBI compared with the general population.^7^ This growing patient burden shows that today's GI fellows will encounter more DGBI cases than previous generations, making robust and standardized DGBI education an urgent priority.^1,7^

Potential Initiatives and Recommendations: Professional GI societies such as the American Gastrointestinal Association (AGA), American College of Gastroenterology (ACG), and European Society of Gastrointestinal Endoscopy (ESGE) recognize the current gaps in education and have begun promoting formal education on DGBI. The Rome Foundation explicitly funds FGIMD training, which now offers 3 annual fellowships in functional disorders, providing protected time and mentorship from experts in the field to cultivate future specialists.^8^ Large academic centers and GI societies (ACG's Functional GI & Motility School, ANMS training programs) have been organizing courses and national meetings specifically for DGBI.

In a recent six-week pilot competency-based curriculum implemented across 6 US fellowships, significant promise was demonstrated.^3^ The results suggest that dedicated DGBI modules can be highly effective when implemented appropriately.^3^ However, such efforts must be scaled nationally and paired with cultural change in training, emphasizing not only core knowledge but also empathy-centered communication skills.

Accreditation bodies should also track measurable DGBI training metrics (such as teaching hours, supervised clinic sessions, and documented competencies), integrating these benchmarks into national training standards.

In any training program, the values and behaviors modeled by educators critically shape attitudes toward patients.^9^ Hence, educators must model positive communication and actively address personal bias. A recent study advocates for a formal faculty development curriculum to train educators to be excellent clinical teachers and provide intentional mentoring that ensures patient concerns are validated rather than minimized or dismissed.^1,9^

Program Directors (PDs) are the cornerstone of implementing DGBI educational strategies at the program level. Integration of DGBI education into fellowship curricula, by didactic lectures, case conferences, and clinical rotations with experts should be standardized to ensure each fellow gains competency in diagnosis and biopsychosocial management.^2,3^ Adoption of validated teaching modules and competency-based assessments may be a valuable resource for PDs. Encouraging trainee research projects on DGBI may spark interest and strengthen understanding. Structured workshops or lectures, either internally or with GI societies, involving a multidisciplinary team of GI psychologists/psychiatrists, dietitians, and pharmacists may build trainee confidence in managing DGBI.^3,5^ Furthermore, PDs should support trainee opportunities for external electives or advanced fellowships at large academic centers, as flexibility for such rotations is strongly desired by both trainees and program leaders.^5^ Enhancing educational experiences through role-playing simulations, GI behavioral medicine rotations, and regular structured feedback on patient interactions can reinforce communication skills while reducing the frustration and fatigue trainees commonly experience when caring for DGBI patients.^9^

At the faculty level, funding protected time for DGBI education and research may help cultivate local experts and expand institutional resources. Acknowledgment through awards at local or national conferences and faculty research grants can foster academic interest. Fostering faculty-fellow collaboration not only enhances trainee education but also motivates faculty to remain engaged with current research and best practices. Finally, implementation of train-the-trainer programs to support faculty who are less familiar with DGBI management is essential to shifting institutional culture and improving long-term patient care.^5,9^

In the future, the prevalence of DGBI is projected to increase worldwide. This serves both as an opportunity and a challenge. Implementing changes in DGBI education is of paramount importance and requires commitment at the institutional, national, and international levels. GI societies worldwide must lead these changes and revise core GI training curricula to explicitly include DGBI competencies. Accreditation bodies should systematically track key DGBI training metrics such as dedicated teaching hours, supervised clinic sessions, and documented competencies to ensure consistency and accountability across programs. Conferences and journals should continue to highlight best practices for functional GI disorders. With anticipated workforce shortages and the growing global burden of DGBI, early and comprehensive training in this area is a strategic investment that will translate into improved patient outcomes and better prepare the next generation of gastroenterologists for the gut-brain era.

## DISCLOSURES

Author contributions: Substantial contributions to the conception or design of the work; or the acquisition, analysis, or interpretation of data for the work: S. Sahu, H.R. Bharadwaj, D.S. Dahiya. Drafting the work or reviewing it critically for important intellectual content: S. Sahu, H.R. Bharadwaj, S. Khan, H. Ali, D.S. Dahiya. Final approval of the version to be published: S. Sahu, H.R. Bharadwaj, S. Khan, H. Ali, D.S. Dahiya. Agreement to be accountable for all aspects of the work in ensuring that questions related to the accuracy or integrity of any part of the work are appropriately investigated and resolved: S. Sahu, H.R. Bharadwaj, S. Khan, H. Ali, D.S. Dahiya. D.S. Dahiya is the article guarantor.

Financial disclosure: None to report.

Informed consent was obtained for this case report.

## ABBREVIATIONS:

ACG: American College of Gastroenterology
AGA: American Gastrointestinal Association
ANMS: American Neurogastroenterology and Motility Society
DGBI: disorders of gut-brain interaction
ESGE: European Society of Gastrointestinal Endoscopy
FGIMD: Functional Gastrointestinal and Motility Disorders
GI: gastroenterology
IBS: irritable bowel syndrome
PD: program directors
UK: United Kingdom
US: United States
